# Efficacy of Enfortumab Vedotin After Chemotherapy With Anti-programmed Death-Ligand 1 (PD-L1) Maintenance Treatment in a Clear Cell Variant of Invasive Urothelial Carcinoma of the Renal Pelvis: A Case Report

**DOI:** 10.7759/cureus.55766

**Published:** 2024-03-08

**Authors:** Hiroshi Hirata, Yoshinobu Hoshii, Hideaki Ito, Toyonori Tsuzuki, Koji Shiraishi

**Affiliations:** 1 Department of Urology, Yamaguchi University, Yamaguchi, JPN; 2 Department of Urology, Graduate School of Medicine, Ube, JPN; 3 Department of Diagnostic Pathology, Yamaguchi University Hospital, Ube, JPN; 4 Department of Surgical Pathology, Aichi Medical University, Nagakute, JPN

**Keywords:** upper urinary tract cancer, pdl1, nectin-4, enfortumab vedotin, clear cell urothelial carcinoma

## Abstract

Among upper urinary tract urothelial carcinoma (UUTUC) cases, there are few reports of the clear cell variant. Systemic chemotherapy will be given according to the usual treatment for urothelial cancer unless lymph nodes or organ metastases make surgical treatment inappropriate. Here, we report a clear cell variant of UUTUC of the left renal pelvis with aortic lymph node metastasis. The patient in this case was treated with systemic chemotherapy, anti-programmed death-ligand 1 (PD-L1) maintenance treatment, radiation therapy, and enfortumab vedotin (EV) therapy. To determine which of the treatments contributed to the therapeutic effect, immunostaining was used. The results indicated that Nectin-4 was expressed in clear cell variant tissues, while programmed cell death protein 1 (PD-1) and PD-L1 expression levels were weak in these tissues. The patient maintained complete remission with these treatments. Two years after the initial treatment, the patient was still alive with no progression or metastasis.

## Introduction

Urothelial carcinoma is a type of urinary tumor that can occur in the upper urinary tract or the lower urinary tract. Upper urinary tract urothelial carcinoma (UUTUC) is a rare genitourinary cancer accounting for 5-10% of all urothelial carcinoma and includes tumors of the renal pelvis and ureter [1,2]. There is no validated biomarker for urothelial carcinoma itself, and naturally, there is no biomarker for clear cell variants. Programmed cell death protein 1 (PD-1) is expressed on the surface of T cells, and cancer cells gain immune escape by expressing its programmed death-ligand 1 (PD-L1); PD-1 expression is associated with poor prognosis, and therefore, immune checkpoint inhibitors, anti-PD-1, and PD-L1 antibodies are being used as novel therapeutic agents in urothelial cancer. Surgery is preferred for the treatment of non-metastatic UUTUC. Overall UUTUC incidence rates decreased from 1.3 to 1.0 cases per 100,000 per year. However, metastatic UUTUC cases are increasing [3]. Based on recent reports, patients with UUTUC with variant histology, such as squamous differentiation, glandular differentiation, etc., had a poor prognosis compared with pure UUTUC [4-6]. We have also shown that UUTUC with variant histology was significantly associated with advanced pathological T stage, more lymphovascular invasion, and worse disease-specific survival compared with pure UUTUC [4]. According to European Association of Urology (EAU) guidelines, if there is variant histology, the UUTUC is classified as a “high-risk UUTUC.” In particular, clear cell variants have only been reported in 14 cases of bladder cancer so far and in fewer cases of upper urinary tract tumors [7]. In this study, we report a case of a metastatic clear cell variant of UUTUC in a renal pelvic tumor treated with systematic GC following an immune checkpoint inhibitor, avelumab maintenance treatment, and enfortumab vedotin (EV) treatment as a third-line treatment. Palliative radiotherapy was also given to L2 spinal metastasis. Two years after the initial treatment, the patient was still alive with no progression or metastasis.

## Case presentation

**Figure 1:**
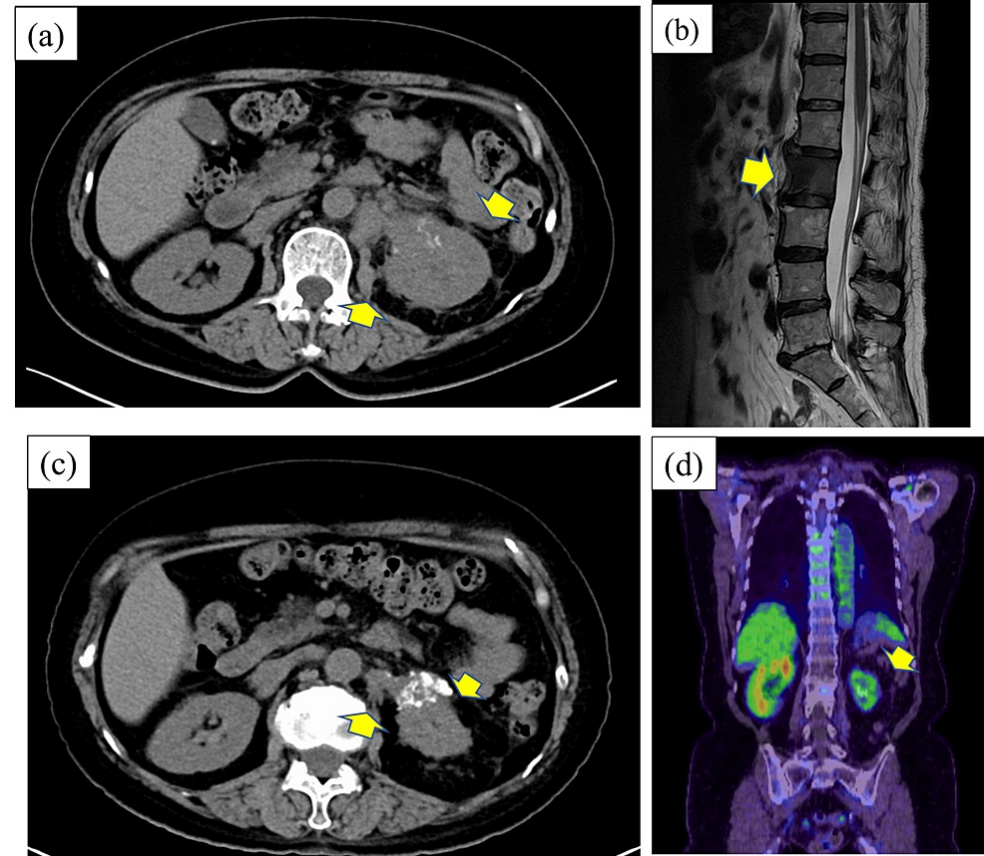
Computed tomography (CT), MRI, and PET-CT imaging. (a) Before treatment, CT scans revealed an oligemic mass in the left renal pelvis and an increased density of surrounding fatty tissue in the renal portal area, showing invasion of the tumor into the fat tissues. Periaortic lymph node metastasis was also noted (cT3N2M0). (b) MRI showed spinal metastasis of the clear cell variant into L2. (c) The tumor volume was significantly decreased after enfortumab vedotin (EV) second-line treatment. (d) PET-CT showed that FDG uptake was weak in the left kidney. Because the uptake was significantly low, this indicated that the tumor viability was suppressed by the treatments. The FDG accumulation in the surrounding lymph nodes was also extremely low. FDG: fluorodeoxyglucose.

The patient was a 62-year-old female and had left flank pain. Since the symptoms persisted, the patient was introduced to our hospital by a medical practitioner. Out laboratory studies revealed a white blood cell (WBC) count of 5.32 x 10^3^/uL (normal 3.3-8.6 x 10^3^/uL), hemoglobin level of 12.8 g/dL (normal 11.6-14.8 g/dL), C-reactive protein (CRP) level of 0.23 mg/dL (normal 0.00-0.14 mg/dL), and serum creatine level of 0.72 mg/dL (normal 0.46-0.79 mg/dL). A tumor biopsy was performed to determine if the tumor was a renal pelvic tumor or a renal tumor. The pathological analysis suggested invasive urothelial carcinoma (UC) and a clear cell variant, G3, from the immunostaining results. Therefore, surgery was not indicated, and systemic chemotherapy using gemcitabine and cisplatin (GC) was initially performed, as it is a regular urothelial cancer treatment approach. After four courses of GC chemotherapy, the original tumor site showed stable disease status. Avelumab, an anti-programmed death-ligand 1 (PD-L1) antibody used for advanced or metastatic urothelial carcinoma, was then administered as a maintenance therapy for eight cycles. The size of the original tumor became gradually increased and the treatment was judged clinically to be ineffective. We next planned EV therapy as a third-line treatment. The patient began experiencing back pain, and an MRI scan showed a new L2 spinal metastasis (Figure 1b). Before EV treatment, palliative radiation therapy (3 Gy/day, total of 30 Gy) was given to improve the painful bone metastasis. This relieved her pain, and EV treatment was then initiated. About three months after beginning this third-line treatment, peripheral neuropathy (grade 3) was observed. The treatment was, therefore, terminated after three courses. Fortunately, a follow-up CT scan showed that the primary tumor and periaortic lymph nodes had gradually shrunk (Figure 1c). PET-CT showed fluorodeoxyglucose (FDG) uptake was weak in the left kidney. Because the uptake was significantly low, this indicated that tumor viability was suppressed by the treatment. FDG accumulation in surrounding lymph nodes was also extremely low (Figure 1d).

**Figure 2:**
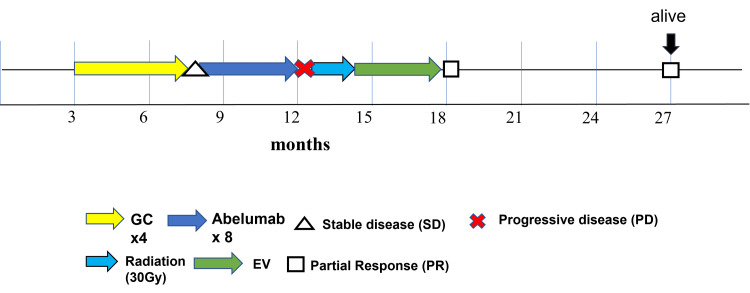
Swimmer plot describing the clinical course of this case. Initially, systemic gemcitabine and cisplatin (GC) therapy was performed for four cycles, and then stable disease status was observed. Avelumab, an anti-programmed death-ligand 1 (PD-L1) antibody, was given as maintenance treatment for eight cycles. Tumor size progressed, and back pain appeared. An MRI scan showed L2 spinal metastasis. Radiation therapy (3 Gy/day, total of 30 Gy) was performed to improve the painful bone metastasis. After the pain was relieved, enfortumab vedotin (EV) therapy was initiated as a second-line treatment. Two years after the initial treatment, the patient was still alive with no progression or metastasis.

The clinical course is shown in Figure 2. After the initial treatment, the patient has had no progression or metastasis so far (Figure 2).

**Figure 3:**
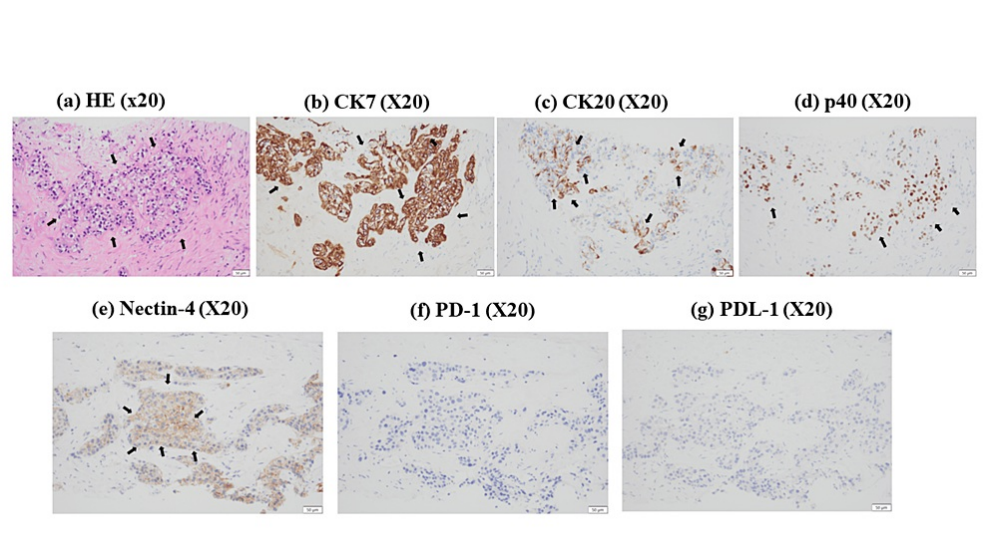
Immunohistochemistry (IHC) analysis of tumor biopsy specimens. (a) Hematoxylin and eosin (H&E) staining showed circular polygonal cells consisting of markedly irregularly swollen nuclei of unequal size with increased chromatin and aqueous clear or acidophilic sporulation with vascular stroma, indicating clear cell carcinoma. (b) Cytokeratin 7 (CK7), (c) CK20, and (d) p40 showed positive IHC staining. These results showed that the clear cell variant was derived from urothelial carcinoma. IHC results indicated that (e) Nectin-4 was expressed on the plasma membrane of the clear cells, but (f) programmed cell death protein 1 (PD-1) nor (g) programmed death-ligand 1 (PD-L1) were expressed in the clear cells.

We additionally investigated the protein expression levels of several antigens, including programmed cell death protein 1 (PD-1), PD-L1, and Nectin-4, in the biopsy specimens. As shown in Figure 3, PD-1 and PD-L1 expression levels were both very low, while Nectin-4 levels were high in the cancerous tissue. 

## Discussion

Two-thirds of upper urinary tract tumors are invasive, with metastases reportedly observed at diagnosis in approximately 9% of cases [1,2]. The majority of upper urinary tract tumors are UC, and non-urothelial histology is rare. Upper urinary tract UCs with different subtypes are high-grade and have a worse prognosis compared with pure UC cases [3-7]. A PubMed-based search for clear cell variant upper urothelial carcinoma yielded four case reports, all of which were case reports without metastases at diagnosis (Table 1) [8-11].

**Table 1:** Reports of a clear cell variant of urothelial carcinoma of the upper urinary tract.

No	Year	Age	Gender	Location	Stage	Treatment	Outcome	Reference
1	1997	70	F	Ureter	TxNxMx	Operation	Alive after six months	[8]
2	2006	58	F	Ureter	T2N0M0	Operation	Alive after 16 months	[9]
3	2015	59	M	Ureter	T2N0M0	Operation	Alive after six months	[10]
4	2021	74	F	Ureter	T2N0M0	Operation, RT, chemotherapy	Recurrence after 22 months; death 34 months after surgery	[11]

This case is the first report of a patient with metastatic clear cell variant UC who has been first treated with systemic chemotherapy and maintained complete remission (CR) with tertiary EV treatment with confirmed Nectin-4 expression. Differential diseases include the clear cell subtype of upper urinary tract adenocarcinoma and metastases of renal cell carcinoma and prostate cancer [11]. Because the case presented here involves a female, prostate cancer is unlikely. Cytokeratin 7 (CK7), CK20, and p40 immunostaining were performed to differentiate between UC and renal cell carcinoma. Our patient’s samples showed positive staining for CK7, CK20, and p40; therefore, ruling out clear cell renal cell carcinoma (Figures 3b-3d). Thus, systemic chemotherapy was used as a treatment for metastatic urothelial carcinoma because our clear cell is a clear cell variant of a variant of urothelial carcinoma, not adenocarcinoma as in renal cell carcinoma. The patient was initially treated with conventional GC (four cycles), followed by avelumab maintenance therapy. However, bone metastasis was then found. EV treatment was performed as a third-line therapy. As of this report, this treatment approach has been successful, with no recurrence observed in the patient. Because there have been no reports in the literature on Nectin-4 expression in the clear cell variant of UC, we used immunostaining to determine why EV treatment was successful. Our results suggest that in the clear cells, Nectin-4 was expressed on the plasma membrane (Figure 3e), while PD-1 nor PD-L1 was expressed (Figures 3f, 3g). A recent case report described a patient with a clear cell variant in the upper urinary tract who was treated with chemotherapy followed by pembrolizumab administration, but the disease rapidly developed distant metastasis, and the patient died [9]. Our report here is the first to examine Nectin-4, PD-1, and PD-L1 expression in the UC clear cell variant and show disease control using EV treatment. Additional cases need to be examined to determine the full efficacy of these treatments, but our case suggests that the clear cell variants are similar to regular UC and may benefit from EV treatment if Nectin-4 is expressed in the clear cell variant tissues.

## Conclusions

Upper urothelial tumors usually have a poor prognosis, with many cases already metastasized at diagnosis, and even a poorer prognosis in the case of variants. In our case, the clear cell type variant is rare and often difficult to treat, but the tertiary treatment EV was extremely successful after GC and avelumab treatment. Therefore, as an interest, we believe that it is crucial to confirm the expression of Nectin-4, the target of EV, in order to predict therapeutic response for metastatic urothelial variant patients as well as for the treating medical staff.
